# Evidence for short duration of antibiotic treatment for non-severe community acquired pneumonia (CAP) in children — are we there yet? A systematic review of randomised controlled trials

**DOI:** 10.15172/pneu.2014.4/432

**Published:** 2014-12-01

**Authors:** Shalom Ben-Shimol, Varda Levy-Litan, Oana Falup-Pecurariu, David Greenberg

**Affiliations:** 13 Pediatric Infectious Disease Unit, Soroka University Medical Center, Faculty of Health Sciences, Ben-Gurion University of the Negev, Beer Sheva, Israel; 23 University Children’s Hospital, Faculty of Medicine, Transilvania University, Brasov, Romania

**Keywords:** community acquired pneumonia, duration of treatment, children, antibiotic treatment, short treatment

## Abstract

*Context*: The ideal duration of antibiotic treatment for childhood community acquired pneumonia (CAP) has not yet been established. *Objective*: A literature search was conducted to evaluate the efficacy of shorter than 7 days duration of oral antibiotic treatment for childhood non-severe CAP. *Data sources*: A systematic literature search was performed using the PubMed database. The search was limited to randomised controlled trials (RCTs) conducted between January 1996 and May 2013 in children up to 18 years old. Search terms included pneumonia, treatment, duration, child, children, days, short, respiratory infection and non-severe (nonsevere). *Study selection*: Only RCTs of oral antibiotic treatment for non-severe CAP in children were included. *Data extraction*: Independent extraction of articles was done by 3 authors using a preformed questionnaire. *Data synthesis*: Eight articles meeting the selection criteria were identified: 7 from 2 developing countries (India and Pakistan), and 1 from a developed country (The Netherlands). Studies from developing countries used the World Health Organization clinical criteria for diagnosing CAP, which includes mainly tachypnoea. None of those studies included fever, chest radiography or any laboratory test in their case definition. The Dutch study case definition used laboratory tests and chest radiographies (x-rays) in addition to clinical criteria. Five articles concluded that 3 days of treatment are sufficient for non-severe childhood CAP, 2 articles found 5 days treatment to be sufficient, and one article found no difference between 3 days of amoxicillin treatment and placebo. *Conclusions*: The efficacy of short duration oral antibiotic treatment for non-severe CAP in children has not been established in developed countries. Current RCTs from developing countries used clinical criteria that may have failed to appropriately identify children with true bacterial pneumonia necessitating antibiotic treatment. More RCTs from developed countries with strict diagnostic criteria are needed to ascertain the efficacy of short duration oral antibiotic treatment for non-severe CAP in children.

## 1. Introduction

Pneumonia is a major cause of morbidity and mortality in children worldwide, with an estimated 2 million deaths of children <5 years of age, annually [1]. Many pathogens are responsible for community acquired pneumonia (CAP) in children, most prominently viruses and bacteria, and determination of specific aetiology is often not possible [1, 2]. Thus, in most guidelines, recommendations for CAP treatment include the administration of antibiotic to the sick child [1, 2, 3]. This therapeutic approach derives from the understanding that most morbidity and mortality associated with CAP caused by bacterial pathogens (e.g. *Streptococcus pneumoniae*) could be significantly reduced if an appropriate antimicrobial agent is administered [2].

Definitions of pneumonia vary widely, from studies in developing populations using the World Health Organization (WHO) clinical criteria for pneumonia diagnosis often requiring only certain respiratory signs or symptoms (mainly tachypnoea) [2], to the requirement of infiltrates on a chest radiograph, combined with high fever, respiratory distress and several laboratory parameters (especially leukocytosis), as customary in most developed countries [1, 3, 4].

Antibiotic treatment recommendations for CAP in children are based on clinical signs and symptoms, patient’s age, disease severity, aetiological diagnosis and epidemiological factors, and require the use of an effective antibiotic given in adequate doses for an appropriate duration [4, 5]. Different antibiotics are being recommended, such as amoxicillin, co-trimoxazole and macrolides [1, 2, 3]. Duration of ambulatory oral antibiotic treatment for non-severe childhood CAP has not been well established. Most guideline recommendations in developed countries are based on custom and practice or extrapolating from other respiratory diseases, like pharyngitis [6]. In contrast, in developing populations recommendations regarding duration of treatment are based on clinical trials, relying on the WHO clinical case definition of pneumonia [7, 8, 9, 10, 11, 12].

This lack of uniformity in CAP definitions has been an important hurdle to studying duration of antibiotic treatment, resulting in heterogeneity in treatment recommendations [2, 3, 5, 13]. Current guidelines for treatment of non-severe pneumonia in developing countries include administration of appropriate antibiotics for 3–5 days [2], while American and European recommendations call for 7–10 days of antibiotic treatment [1, 3].

As guidelines for the management of non-severe CAP in children in developing countries allows a shorter than 7–10 days duration of antibiotic treatment, a literature search was conducted to evaluate randomised controlled trials that assessed the efficacy of short duration oral antibiotic treatment against non-severe childhood CAP and whether the current evidence allows a similar approach to be used in populations in developed countries.

## 2. Methods

### 2.1 Data sources and searches

Literature searches were performed using the PubMed database. The PRISMA reporting guidelines were followed conducting this systematic review [14]. Only articles written in English were reviewed. Five different searches were conducted, limiting searches to articles published between 1 January 1996 and 1 May 2013, using a combination of selected words.

### 2.2 PubMed search strategy

The following key words were used in 5 literature searches:

- Search no. 1 — ‘pneumonia’, ‘treatment’, ‘duration’, ‘child’ and ‘days’.
- Search no. 2 — ‘pneumonia’, ‘treatment’ and ‘short’.
- Search no. 3 — ‘respiratory infection’, ‘treatment’, ‘short’, ‘duration’ and ‘child’.
- Search no. 4 — ‘pneumonia’, ‘children’, ‘treatment’ and ‘non-severe’.
- Search no. 5 — the same key words used in search no. 4 were used, with one change: instead of the word “non-severe” the word ‘nonsevere’ was used.

### 2.3 Selection criteria

The following predetermined inclusion criteria were used: (i) RCTs evaluating the duration of oral antibiotic treatment shorter than 7 days in children with non-severe CAP; (ii) study population of children aged 0–18 years with non-severe CAP; (iii) studies comparing different treatment durations of the same antibiotic regimen; (iv) studies comparing different drugs or comparing antibiotic regimen with placebo; (v) studies published in English; (vi) type of outcome measure (including treatment failure and cure rate). All non-RCT publications (e.g. meta-analyses, reviews etc.), RCT in adults and RCT where non-oral (intravenous, [IV]) treatment was given, were excluded.

### 2.4 Study selection and data extraction

All articles identified in the primary search were screened independently for relevancy by 3 of the authors (SB, DG, VL) who read all article titles and abstracts in a standardised manner. Disagreements between reviewers were resolved by discussion and consensus reached. A preformed questionnaire form was used by the 3 reviewers to extract the data evaluating study population (including age, ambulatory or hospital setting), sample size, randomisation, case definition of CAP, disease severity, antibiotic treatment (including type, dose, administration method and duration of treatment) and outcome. Identified relevant articles were further evaluated for inconsistencies by 3 authors (SB, DG, VL) as was the case with articles without available abstracts. Disagreements were resolved by discussion between the 3 reviewer authors. Additionally, all of the articles that were eligible for inclusion were manually searched for relevant references.

### 2.5 Data items

Information was extracted from each selected RCT on: (i) study location (developing or developed country); (ii) characteristics of trial participants (including number and age); (iii) antibiotic regimens (including type of drug, administration method and duration of treatment) given; (iv) outcome (cure/failure rate).

### 2.6 Evaluation of study quality

No validated instrument was used to assess the quality of the RCTs. However, all 8 RCTs evaluated in our search were evaluated for the following data to assess bias possibilities, including: (i) hospital versus community based studies; (ii) identification of aetiology (pathogen), (iii) duration of follow up; (iv) co-morbidities (e.g. asthma); and (v) adherence evaluation.

### 2.7 Data synthesis and analysis

No meta-analysis was performed due to insufficient number of comparable estimates. Findings of studies were described and synthesised in a narrative format.

## 3. Results

### 3.1 Eligible studies

**Figure 1:**
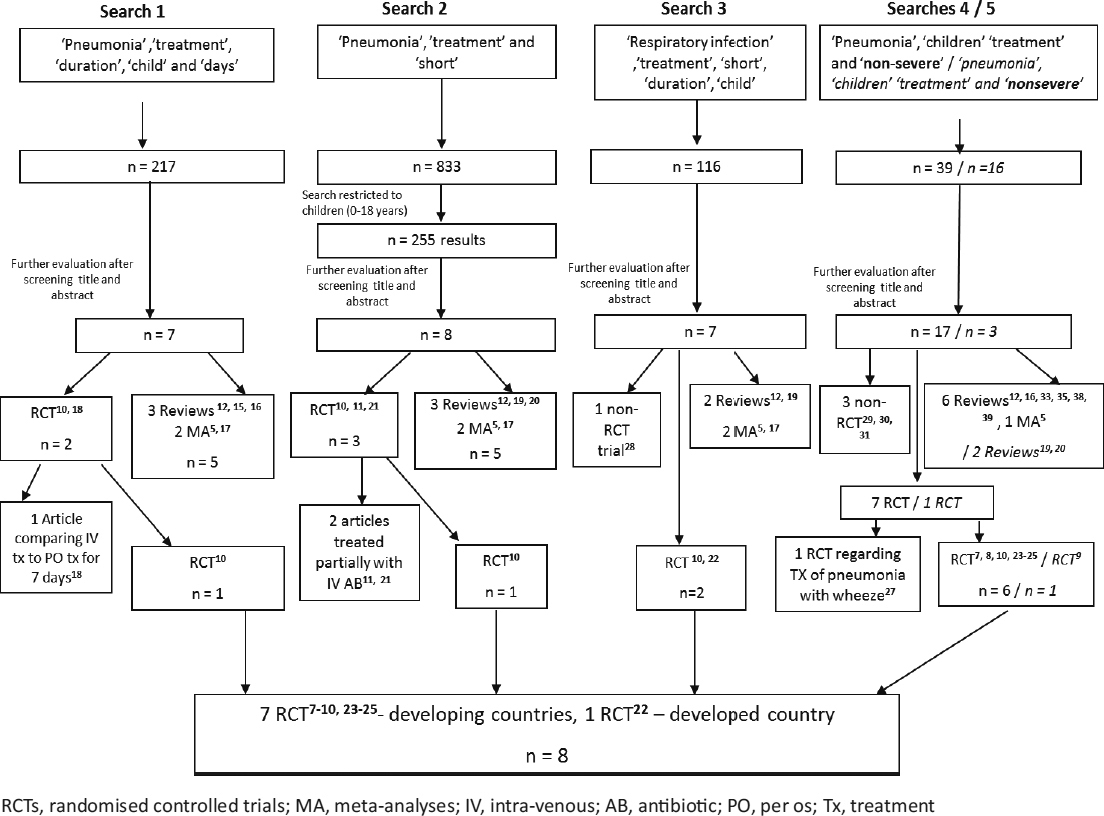
Literature searches 1–5, performed in Medline using PubMed, for short duration treatment of childhood CAP

In the first search, 217 articles were found (Figure 1) and further evaluated by reading all titles and abstracts. Seven articles were identified: 3 articles were reviews [12, 15, 16], 2 were meta-analyses [5, 17], and 2 were RCTs [10, 18]. One RCT was excluded since it compared intravenous to oral antibiotic treatment [18]. In the second search, 833 articles were identified. After restricting those results to the paediatric population (0–18 years), 255 articles were found. Further evaluation (using the preformed questionnaire form) identified 8 articles: 2 of them were meta-analyses [5, 17], 3 were reviews [12, 19, 20], and 3 were RCTs [10, 11, 21], of which, 2 were regarding pneumonia treated partially with intravenous antibiotics [11, 21]. The third, the fourth and the fifth searches identified one RCT [10] which met the inclusion criteria and was included in the systematic review and 2 meta-analyses [5, 17] identified in previous searches, while an additional 7 RCTs [7, 8, 9, 22, 23, 24, 25] that met the inclusion criteria were identified, 4 non-RCTs [28–31] and 4 more reviews [33, 35, 38, 39].

### 3.2 Study characteristics

Table 1 summarises the main characteristics of the included studies. Eight articles meeting the selection criteria were identified [7, 8, 9, 10, 22, 23, 24, 25], 7 of them were RCTs from developing countries (India and Pakistan) and 1 was from a developed country (The Netherlands). The participants, interventions, comparators, outcomes and study designs (PICOS) characteristics [14] were as follows: Patient’s age was 2–59 months in all 8 studies and the number of patients varied between 873 and 2,188 in all 7 studies from developing countries, compared with 118 patients in the Dutch study. Of the 7 studies from developing countries, 2 compared short durations of 3 versus 5 days of treatment with the same drug (amoxicillin) [8, 10], 1 compared 3 days of treatment with amoxicillin versus placebo [9], 1 compared standard versus double dose of amoxicillin for 3 days [7], 1 compared 3-day amoxicillin versus 5-day co-trimoxazole [23], 1 compared standard versus double dose of co-trimoxazole for 5 days [24], and 1 compared co-trimoxazole versus amoxicillin, both for 5 days [25]. The Dutch study compared 3 days treatment with azithromycin to 10 days treatment with co-amoxiclav (amoxicillin/clavulanic acid) [22].

**Table 1:** Main characteristics of randomised controlled trials meeting selection criteria for short duration of antibiotic treatment of non-severe CAP in children

Study (year) [Reference]	Country	Patient’s age	No. of paients	Regimens compared	Case definition	Study results (cure rate) *p* value
Hazir et al. 2007 [7]	Pakistan	2–59 months	876	3 days amoxicillin (standard dose) vs. 3 days amoxicillin (high dose)	WHO^a^	97.0% vs. 95.9% (*p* = 0.55)
Agarwal et al. 2004 [8]	India	2–59 months	2,188	3 days amoxicillin vs. 5 days amoxicillin	WHO	89.5% vs. 89.9% (*p* = 0.78)
Hazir et al. 2011 [9]	Pakistan	2–59 months	873	3 days amoxicillin vs. placebo	WHO	92.8% vs. 91.7% (*p* = 0.60)
MASCOT 2003 [10]	Pakistan	2–59 months	2,000	3 days amoxicillin vs. 5 days amoxicillin	WHO	79% vs. 80% (*p* = 0.74)
Awasthi et al. 2008 [23]	India	2–59 months	2,009	3 days amoxicillin vs. 5 days co-trimoxazole	WHO	86% vs. 90% (*p* = 0.24)
Rasmussen et al. 2005 [24]	Pakistan	2–59 months	1,143	5 days co-trimoxazole (standard dose) vs. 5 days co-trimoxazole (high dose)	WHO	80.6% vs. 78.8% (*p* = 0.46)
Catchup 2002 [25]	Pakistan	2–59 months	1,459	5 days amoxicillin vs. 5 days co-trimoxazole	WHO	83.9% vs. 81.1% (*p* = 0.17)
Ferwerda et al. 2001 [22]	The Netherlands	3 months–12 years	118	3 days azithromycin vs. 10 days co-amoxiclav	CXR or clinical signs^b^	91% vs. 87% (*p* = 0.55)

MASCOT, Pakistan Multicentre Amoxycillin Short Course Therapy; WHO, World Health Organization; CXR, Chest radiograph (X-ray)

^a^high respiratory rate adjusted for age

^b^fever or leukocytosis or rhonchi

### 3.3 Case definitions

All 7 trials from developing countries [7, 8, 9, 10, 23, 24, 25] evaluated children at the age of 2–59 months and used the WHO clinical definition for non-severe CAP, including tachypnoea (high respiratory rate adjusted for age) and excluding severe (defined as lower chest in-drawing) and very severe diseases. None of those studies included fever, chest radiography or any laboratory test in their case definition. In contrast, 1 RCT from the Netherlands [22] evaluated children at the age of 3 months to 12 years, and defined lower respiratory tract infection (LRTI) as the presence of respiratory signs and symptoms in combination with consolidation on a chest radiograph or clinical evidence of LRTI (including fever, leukocytosis, rales, rhonchi or signs of consolidation on physical examination).

### 3.4 Risk of bias within studies

The following bias points were recognised in the systematic search: (i) The settings were of outpatient departments [7, 10], hospital patients [8, 9, 22] community-based studies [23], and both outpatient departments and community based [24, 25]. (ii) Identification of aetiology (pathogen) was only performed in the Dutch study [22]. (iii) Duration of follow up varied between 7 days [24, 25], 15 days [7, 8, 9, 10, 23] and 30 days [22]. (iv) Children with asthma or recurrent (>3 episodes) wheezing co-morbidity were excluded in the 7 studies from developing countries [7, 8, 9, 10, 23, 24, 25], but in the Dutch study, children with asthma were included [22]. (v) Adherence was evaluated in all 8 RCTs.

### 3.5 Efficacies of shorter than 7 days duration of oral antibiotic treatment for childhood non-severe CAP (Table 1)

Of the 7 articles from developing populations, 4 articles concluded that 3 days of oral amoxicillin are sufficient treatment for non-severe childhood CAP [7, 8, 10, 23], 2 articles found 5 days treatment to be sufficient, either with co-trimoxazole [24] or amoxicillin [25], and 1 article found no difference in the clinical outcome between 3 days of amoxicillin treatment and placebo [9].

In all these 7 studies, there were no statistically significant differences in cure rate (or failure rate) between the regimens evaluated, including comparison of: 3 days of amoxicillin in different dosages (high versus standard dose), both achieving cure in >95% of the patients [7]; 3 versus 5 days of amoxicillin, achieving cure in >89% [8] and ≥79% [10] of the patients; 3 days of amoxicillin versus 5 days of co-trimoxazole, both achieving cure in ≥86% of the patients [23]; 5 days of amoxicillin versus 5 days of co-trimoxazole, both achieving cure in >81% of the patients [25]; 5 days of co-trimoxazole in different dosages (high versus standard dose), both achieving cure in >78% of the patients [24]; and 3 days of amoxicillin versus placebo, both achieving >91% cure rate [9].

The one trial from a developed country (The Netherlands) found 3 days of azithromycin to be an effective treatment (91% cure rate) compared to 10 days of co-amoxiclav (87% cure rate) [22].

### 3.6 Articles not meeting the selection criteria (Figure 1)

An additional 3 RCTs, which compared durations of treatment of intravenous antibiotics for childhood pneumonia, were identified. These were RCTs from both developed and developing countries [11, 18, 21]. Of those trials, one suggested that 7 days of oral amoxicillin treatment is effective (and equivalent to IV penicillin) for non-severe pneumonia [18], 1 found 5 days of oral amoxicillin to be effective in the treatment of severe pneumonia [11], and 1 trial showed similar efficacy for 4 days compared with 7 days IV treatment of bacterial infections, including bacterial pneumonia [21].

Other articles included 1 RCT in adults [26], 1 RCT regarding treatment of pneumonia with wheeze [27], 4 non-RCTs [28, 29, 30, 31], 9 reviews, and 2 meta-analyses [5, 17].

## 4. Discussion

Antibiotic treatment is the cornerstone of bacterial CAP management. However, since it is difficult to ascertain pneumonia aetiology, clinical definitions (in some developed countries combined with laboratory parameters) are often guiding treatment [32]. Moreover, even in cases when antibiotic treatment is recommended, significant variation in treatment duration is notable in published guidelines, ranging from 3 to 10 days. The literature search identified only 8 RCTs addressing the issue of short duration oral antibiotic treatment for non-severe CAP in children. Of those clinical trials, 7 were from 2 developing countries (India and Pakistan) where the criteria for diagnosing CAP are based on the WHO recommendations and include only a few clinical symptoms, mostly tachypnoea. One RCT from a developed country (The Netherlands) was identified [15] comparing short treatment of 3 days versus 10 days treatment. This study did include fever, chest radiography and leukocytosis, as well as respiratory signs and symptoms in its case definition. However, the study compared short treatmentwith azithromycin, a long acting macrolide, and a longer treatment with co-amoxiclav which is a short-acting penicillin. Thus, to our knowledge, there is currently no RCT from a developed country which compared short versus long duration of oral antibiotic treatment using the same drug.

Several reasons make short duration of antibiotic treatment for CAP an appealing option. First, treatment for the shortest effective duration will reduce the overall cost of treatment for both health care systems and the child’s caregivers. Second, short treatment could also improve patients and parents compliance and adherence to the prescribed treatment. Third, drug toxicity and adverse events could be minimised. Fourth, shorter exposure of both pathogens and normal microbiota to antimicrobials will minimise the selection for antimicrobial resistance [1].

In a recent American guideline it was noted that treatment courses of 10 days have been best studied, although shorter courses may be just as effective, particularly for non-severe disease managed on an outpatient basis [1]. Comparative studies from the developing world, using the WHO clinical criteria for pneumonia diagnosis [2], suggested that in children <5 years old the 3 days treatment course is equivalent to 5 or 7 days treatment [5, 7, 8, 12, 18, 33]. However, a recent study, using similar criteria, found no signifcant difference between a 3-day amoxicillin and placebo treatment [9] suggesting that the WHO clinical criteria may not appropriately identify children with true bacterial pneumonia necessitating antibiotic treatment. Indeed, these criteria do not include temperature measurement, peripheral white blood cell count, chest radiographs and cultures, resulting in poor discrimination between acute viral infections (i.e. bronchitis or bronchiolitis) and pneumonia which is likely to be bacterial [34, 35]. Indeed, the WHO clinical case definition is highly sensitive but lacks specificity [36].

British guidelines from 2011 concluded that due to difficulties, mainly regarding the WHO definitions for non-severe pneumonia in the comparative trials conducted in developing countries, it is still not known whether a 3-day antibiotic course is sufficient to treat a child with bacterial pneumonia [3]. Thus, extrapolating from studies conducted in the developing world to developed populations may be impractical and dangerous.

As the WHO case definition for non-severe pneumonia include only ‘cough or difficult breathing with fast breathing’ [2], it is possible that trials using this broad and non-specific definition for CAP will in fact include several other respiratory diseases. Thus, recommendations for CAP treatment worldwide are often based on clinical trials conducted in developing countries, which actually treated a spectrum of respiratory illnesses, with only a minority of those cases being bacterial pneumonia necessitating antibiotic treatment. This possibility was acknowledged by the group from Pakistan, as they wrote: “The main limitation of our study was that we did not have a definitive etiological diagnosis” [10], and “Fast breathing may be caused by conditions other than pneumonia” [9].

This notion was further emphasised in a recent American study [37], concluding:

The WHO criteria demonstrated poor sensitivity for the diagnosis of radiographic pneumonia in a US-based pediatric emergency department…WHO criteria may not be a sensitive screening tool for the diagnosis of pneumonia in children.

Furthermore, the lack of criteria, other than tachypnoea and dyspnoea, in defining pneumonia (i.e. chest radiograph) had led to the absurd notion in one study from India [23] in which the inclusion criteria for children with pneumonia and wheezing were: “…[children] who have persistent fast breathing after nebulisation with salbutamol, and have **normal** chest radiograph.”

It is noteworthy that the WHO criteria for diagnosing pneumonia are first and foremost intended to be used in developing populations where mortality from pneumonia is high. These diagnostic criteria main purpose is to allow for early and rapid treatment of children with suspected pneumonia in settings of low resources for diagnosis (that is, where laboratory testing and chest radiographs are not available) and high mortality. Therefore, it is appropriate that these criteria will have high specificity allowing treatment of all cases while paying the cost of over-treatment. This further emphasises our conclusion that studies relying on these criteria should not be used to dictate treatment duration in different settings in developed populations.

We believe that the current data on non-severe CAP in children does not provide convincing evidence for the ideal duration of oral antibiotic treatment. Only future RCTs with strict disease definitions, including chest radiography, fever and inflammatory markers (e.g. leukocytosis), could lead to sound recommendation for short duration treatment of childhood CAP.

The main limitations of this study lie in the methodology of the literature search. The search was limited to articles written in the English language and identified by the PubMed search engine. As is the case in any systematic literature review, articles which did not use any of the defined search words may have been un-identified. However, all references in all articles meeting selection criteria were evaluated to minimise the possibility of missed relevant trials.

The efficacy of short duration oral antibiotic treatment for non-severe CAP in children has not been established in developed countries. Current RCTs from developing countries used clinical criteria that may have failed to appropriately identify children with true bacterial pneumonia necessitating antibiotic treatment.
